# Appointments and Resignations

**Published:** 1853-08

**Authors:** 


*Appointments and Resignations.*—Dr. Leidy, of Philadelphia, has been elected to the chair of Anatomy in the University of Pennsylvania, made vacant by the death of Dr. Horner.

We learn that Drs. Baxly, Rieves, Edwards, and Cobb, have resigned their respective chairs in the Medical College of Ohio. We have not yet seen an official announcement of their successors.

Dr. R. L. Breckenridge, of Louisville, Ky., has been elected to the chair of Materia Medica, in the Kentucky School of Medicine, *vice* Dr. Foree, resigned.

